# Validity of Coupling TRACAB’s Gen5 and Mediacoach Systems to Calculate Accelerations and Decelerations in Professional Football

**DOI:** 10.3390/s25061804

**Published:** 2025-03-14

**Authors:** Joaquín González-Rodenas, Fabio Nevado, Roberto López-Del Campo, Aitor Soler-Aguinaga, Fidel Agulló, Víctor Moreno-Pérez, Juan Del Coso

**Affiliations:** 1 Sport Sciences Research Centre, Rey Juan Carlos University, 28942 Fuenlabrada, Spain; 2 Department of Competitions, La Liga, 28043 Madrid, Spain; fnevado@laliga.es (F.N.); rlopez@laliga.es (R.L.-D.C.); 3 Strength and Conditioning Department, Elche Club de Fútbol, 03208 Elche, Spain; aitorjsoler@gmail.com (A.S.-A.); fidelagullo@hotmail.com (F.A.); 4 Sports Research Center, Miguel Hernández University of Elche, 03202 Elche, Spain; vmoreno@umh.es; 5 Center for Translational Research in Physiotherapy, Department of Pathology and Surgery, Miguel Hernandez University of Elche, 03550 San Joan, Spain

**Keywords:** soccer, team sport, tracking device, physical performance, accelerometry

## Abstract

This study assessed the agreement between an optical video tracking system (TRACAB Gen5, Chyronhego, NY, USA) coupled with Mediacoach software (*LaLiga*, Spain) and accelerometry data derived from a GPS-Inertial Measurement Unit (IMU) device (GPS-IMU; Hudl, Lincoln, NE, USA) in measuring the number and intensity of accelerations (ACCs) and decelerations (DECs) during professional football matches. Data were collected from 46 *LaLiga* players across three seasons (2021–2024), resulting in 662 comparative match datasets, including 95,371 ACCs and 100,952 DECs recorded by the inertial unit of the GPS-IMU devices (considered as the reference criterion). The video tracking system consistently reported lower values for both ACCs (*p* < 0.001, *d* = 0.47) and DECs (*p* < 0.001, *d* = 1.17) than GPS-IMU. Despite this discrepancy, agreement between methods was very high for total ACCs (R^2^ = 0.954) and DECs (R^2^ = 0.950), as well as for moderate-level ACCs (2–4 m/s^2^, R^2^ = 0.956) and DECs (−2 to −4 m/s^2^, R^2^ = 0.944). The agreement was lower for high-intensity ACCs (>4 m/s^2^, R^2^ = 0.338) and DEC (<−4 m/s^2^, R^2^ = 0.838). In conclusion, integrating the TRACAB Gen5 optical video tracking system with Mediacoach software provides an effective method for assessing the physical load of professional football players. However, acceleration data obtained from this video tracking system should not be used interchangeably with GPS-IMU devices, as it systematically underestimates both accelerations and decelerations.

## 1. Introduction

In the dynamic and physically demanding environment of professional male football, understanding the multifaceted nature of players movement during gameplay is crucial for optimizing performance and reducing injury risk [1,2]. Traditional running metrics such as total distance covered and high-speed running have been deeply analyzed in the existing literature [3,4]. However, this distance-based approach often misses accounting for some high-intensity actions such as quick changes in direction, bursts, or stoppages, which require significant effort but occur over short distances. Additionally, some of these actions involve starting from a stationary or near-stationary position, which limits the ability to reach high peak running speeds. As a result, their peak running speed is typically low, but the physical demands such as muscular force and neuromuscular coordination are still significant [5]. Therefore, today it is well accepted that measuring only running distances at various speed thresholds provides a broad but incomplete overview of players’ external load during matches [6].

In professional football, accelerations (ACCs) and decelerations (DECs) are increasingly acknowledged as vital components of a player’s physical performance for both match and training exposures [7]. Data on high-intensity ACCs and DECs (habitually ACC > 2 m/s^2^ and DEC < −2 m/s^2^) are routinely collected and integrated with running distance metrics to provide a comprehensive understanding of match load [7]. From a performance perspective, these measures offer a nuanced understanding of match load, capturing the intensity and frequency of physically demanding actions that may not be reflected in distance-based metrics alone. This information is critical for coaches and sports scientists in designing training programs that replicate match conditions and for assessing recovery needs [8]. Furthermore, tracking ACCs and DECs is a vital tool for injury prevention, particularly in mitigating the risk of muscle injuries, which are frequently associated with high-intensity actions and rapid changes in momentum [9]

Global Positioning System-Inertial Measurement Unit (GPS-IMU) devices have become the standard for capturing the frequency and intensity of ACCs and DECs during matches, providing valuable insights into external load [10]. These wearable devices, typically secured in a vest, collect real-time data on player movements with high accuracy [9]. However, despite their effectiveness, the requirement for athletes to wear the devices during matches can pose challenges, with some players reporting discomfort or restrictions in their range of motion. An alternative to wearable technology is real-time optical video tracking systems, which are already widely utilized in professional football to calculate running speeds and distances. Recent advancements in optical tracking have made it possible to derive ACC and DEC metrics from video data, offering a non-invasive method for monitoring external load [11].

Specifically, TRACAB’s Gen5 is an optical tracking system used in professional football to capture player movements [12] and physical performance [3]. This system uses advanced algorithms and multiple camera feeds to accurately track players’ movements on the field without requiring additional equipment [13].

However, while the validity and interchangeability of TRACAB’s Gen5 to assess football players’ running distances at different speeds has been tested against other reference criteria such as the VICON motion capture system [13], PROZONE [14], Second Spectrum [15], and GPS-IMU devices [16,17], the validity of these systems to assess the number of ACCs and DECs is limited. To our knowledge, only the study by Pons et al. [18] evaluated the level of agreement between TRACAB and GPS WIMU PRO in ACC/DEC demands in professional football. This study observed that both systems exhibited an acceptable level of agreement, although caution was suggested when interpreting ACC/DEC values above 3 m/s^2^. Specifically, TRACAB recorded slightly higher values than GPS-IMU devices in high-intensity ACCs/DECs, whereas the GPS-IMU device reported slightly higher values in low-intensity ACCs/DECs (<2 m/s^2^).

Despite these findings, further research is necessary to replicate and extend these results into different competitive settings. Stadium-specific factors, such as variations in camera setup, architectural design, and environmental conditions, may influence the accuracy of optical tracking, necessitating additional validation across diverse venues. While TRACAB provides a non-invasive alternative to GPS-based systems, its ability to consistently measure ACC/DEC across varying contexts requires further investigation. By replicating previous research in a new setting, this study strengthens the generalizability of tracking systems, offering valuable insights for sports scientists and performance analysts in elite football. Therefore, the aim of this study was to evaluate the degree of agreement between the number and intensity of ACCs and DECs recorded by an optical video tracking system (TRACAB’s Gen5) coupled with a software that derives ACCs and DEC metrics from video data (Mediacoach) with those measured using accelerometry data derived from the IMU sensor within GPS-IMU devices (WIMU Pro) during official football matches.

## 2. Methods

### 2.1. Participants

Forty-six male professional football players from a team competing in the top division of Spanish football (*LaLiga*) were prospectively followed up over three consecutive seasons (2021–2022, 2022–2023, and 2023–2024). They had a mean ± standard deviation (SD) age = 28.7 ± 3.6 years, body mass = 77.6 ± 6.9 kg, and height = 180 ± 7.3 cm. In the sample, there were 6 external midfielders, 12 external defenders, 9 forwards, 12 central midfielders, and 7 central defenders. Goalkeepers were excluded from the study as their running and ACC/DEC profiles differ from those of the field players. A total of 662 match data points from 46 professional football players were analyzed during the observational period. All players performed 6–8 h of football training and 1–2 competitive matches per week. Before the start of this investigation, an institutional Ethics Review Committee (code: DPC.VMP.240213) approved the procedures included used in this study, following the latest version of the Declaration of Helsinki. In addition, *LaLiga* authorized the use of data on players’ ACC and DEC profiles during official matches, and the study does not contain information that identifies players, as per *LaLiga’s* ethical guidelines for research.

### 2.2. Study Design

In all official matches of *LaLiga* played at home by the team under investigation (n = 57; 19 per season), players wore a GPS-IMU device (WIMU Pro, Hudl, Lincoln, NE, USA) 10 Hz for GPS data and 1000 Hz for inertial sensor data) to obtain data on ACCs and DECs during match play. Then, ACCs were clustered in groups between 2–4 m/s^2^ and > 4 m/s^2^ using the software associated with the GPS-IMU devices (SPRO, version 2.2.0. Hudl, Lincoln, NE, USA). DECs were also clustered in this case in groups between −2–[−4] m/s^2^ and <−4 m/s^2^. In both cases, the limit for ACCs and DECs was up to +/− 30 m/s^2^. This study selected the thresholds of 2–4 m/s^2^ and >4 m/s^2^ to differentiate between moderate accelerations and high-intensity accelerations, following classifications established in the previous literature on team sports [9]. The data recorded by the GPS-IMU devices were stored in a 16 GB internal flash memory. The device had an internal battery with a four-hour duration, it weighed 70 g, and its dimensions were 81 × 45 × 16 mm. The same matches were video captured by TRACAB’s Gen5 optical video tracking system (Chyronhego, NY, USA), consisting of 16 cameras placed at different positions in the stadium, recording positional data at 25 Hz). At the same time, the data on the tracking system were used to calculate the number of ACCs and DECs using the same categories explained above by using Mediacoach software (https://www.mediacoach.es/) (*LaLiga*, Spain). We used this categorization using Δ2 m/s^2^ groups to assess the utility of coupling TRACAB’S Gen5 and Mediacoach to assess the total number of and the low- and high- intensity ACCs/DECs in male professional football [19]. Data were captured from all players that played the matches, including starters and substitutes that played at least 10 min. All matches were played on outdoor natural grass within a pitch dimension of ~100 × 70 m. The level of agreement between the ACCs and DECs estimated by the video system and those obtained with the GPS-IMU devices, considered as the reference criterion, was analyzed to test the utility of coupling TRACAB’s Gen5 and Mediacoach to assess ACC/DEC profiles in professional football players.

### 2.3. ACCs and DECs Obtained with GPS-IMU Devices

Players’ match running speed and distance, along with the number of ACCs and DECs, were monitored with the WIMU Pro GPS-IMU devices. For this study, we discarded all running metrics, as the validity of the video tracking system to assess distances at different speed thresholds has been already studied. For this study, we only analyzed accelerometer data obtained with the GPS-IMU devices. The device integrates different sensors: four 3D accelerometers operating at different scales: ±16 G, ±16 G, ±32 G and ±400 G; three gyroscopes, two at ±2000°/s at 1000 Hz and one at ±4000°/s at 1000 Hz; a 3D magnetometer ± eight Gauss at 160 Hz; and a barometer ±1200 mbar at 100 Hz. The present study captured accelerometry data derived from the IMU sensor embedded within the GPS-IMU device as the reference criterion for accelerometry data. We set a threshold cut-off of 0.1 m/s^2^ for at least 100 ms to account for any acceleration/deceleration, following previous recommendations [20], and we recorded the highest value of acceleration/deceleration for each complete action [18]. These GPS-IMU devices have shown a good level of accuracy (coefficient of variation < 3.5%) for the measurement of high-speed running movements and ACCs/DECs during football exposure [21].

For this measurement, the GPS-IMU device was positioned inside a vest, and it remained between the participants’ shoulders without hindering the upper body movements. Players wore the GPS-IMU during the warm-up and match exposure to obtain data at 10 Hz for GPS-based variables and at 1000 Hz for inertial sensor data. Each player wore the same GPS-IMU device during the whole season period to avoid inter-device variability [22]. According to the manufacturer’s recommendations, all devices were activated 15 min before the onset of the match to allow for acquisition of satellite signals and synchronization of the GPS clock with the satellite’s atomic clock. Daily, after the end of the match, data obtained with the GPS-IMU devices were downloaded to a personal computer and analyzed using the SPRO software. (Version 2.2.0. Hudl, Lincoln, NE, USA). For this process, a member of the strength and conditioning staff of the team created individual files for each player to obtain data on the match alone by removing data from the warm-up and the pause between match halves. Then, this file was analyzed to obtain running metrics and ACCs and DECs. The software categorized ACCs and DECs into intensity zones based on predefined thresholds, and we used a cluster of data between 2–4 m/s^2^ and >4 m/s^2^ to match those obtained with the TRACAB’s Gen5-Mediacoach coupling. These thresholds were selected by considering previous studies that have analyzed the ACC demands in elite football [9]. When the file obtained from the GPS-IMU device did not include complete match exposure data (such as when it only captured a portion of the match due to disconnection), it was discarded for the statistical analysis.

### 2.4. ACCs and DECs Obtained with Video Tracking

Players’ match running speed and distance were collected by using the TRACAB’s Gen5 optical video tracking system. TRACAB’s Gen5 system consists of a network of 16 high-definition cameras installed around the stadium to cover the entire field. The system has been trained to identify all players on the pitch and the ball and it tracks them individually based on their unique visual signatures. Cameras capture at 25–50 frames per second, ensuring the continuous tracking of every player and the ball. Specifically, the system determined the X, Y (field position), and Z (height) coordinates of each player at 25 Hz in real-time and calculated speed as the rate of change in a player’s position over time. Distance was computed by summing the total displacement of a player’s movements over time, and curved paths were accounted for as the system captures granular position data for non-linear motion. TRACAB’s Gen5 uses filtering algorithms to smooth data and reduce noise caused by occlusions or overlapping players (e.g., during corner kicks). In *LaLiga*, data provided by TRACAB’s Gen5 in each match are further computerized by a custom-made software called Mediacoach that allows for calculation of the match running data and more specific variables such as sequences [23], playing style [24], team formations [25], and complex tactical variables [26]. Mediacoach also allows for the data correction of TRACAB’s Gen5 data on overlaid players’ coordinates, as there is an operator for each match that visually corrects the situations in which the positioning coordinates are erroneous because they move away from the position of the player to whom the data belong. This means the TRACAB’s Gen5 video tracking data are more refined with Mediacoach. Taking into account this correction, real-time positional TRACAB’s Gen5 data were used by Mediacoach to derive ACCs as the rate of change in speed over time. Hence, coupling TRACAB’s Gen 5 system and Mediacoach ensures calculations of instantaneous speed and the resulting ACCs and decelerations. As for the GPS-IMU devices, we set a threshold cut-off of 0.1 m/s^2^ for at least 100 ms to account for any acceleration/deceleration, and, if there was more than one value above the threshold in the same acceleration curve, we retained the highest value as the acceleration value for the given action. Football-specific running metrics measured using TRACAB’s Gen 5 system were validated against reference criteria [13], and the calculations applied by Mediacoach to calculate running metrics have also been validated against GPS-IMU devices [16,17].

### 2.5. Statistical Analyses

Data on ACCs and DECs per player and per match were downloaded and analyzed in each specific software (SPRO for GPS-IMU data and Mediacoach for video tracking data) and then converted into a .csv file for statistical analysis. The files were then merged to compare the data of the two methods under investigation using the Statistical Package for Social Sciences software (SPSS, version 28.0; SPSS Inc., Chicago, IL, USA). The level of agreement between the ACC and DEC variables was analyzed by calculating several statistics. Differences between methods were analyzed using the coefficient of variation. Paired-sample t-tests were conducted to assess whether the differences in all variables measured with GPS-IMU and video tracking reached statistical significance. The Cohen’s *d* for paired samples was calculated as an effect size for each data comparison and interpreted as <0.2, trivial; 0.2–0.6, small; 0.6–1.2, moderate; 1.2–2.0, large; 2.0–4.0, very large; and >4.0, extremely large. The root mean square error (RMSE), the standard error of estimate (SEE), the typical error of estimate (TEE), and the standardized mean bias (SMB) were calculated to quantify the average magnitude of error between the measured (video tracking) and reference values (GPS-IMU). Additionally, the coefficient of determination (R^2^), the intraclass correlation coefficient (ICC; two-way random), and the limits of agreement (LoA) between measurements were also computed to catalog the agreement between measurements. Data on each variable are presented as mean ± SD, including minimum and maximum values (range). Statistical significance was reported to indicate the strength of the evidence (*p* < 0.050).

## 3. Results

A total of 662 match data points from 46 professional football players were analyzed during the observational period. There was a total of 95,371 ACCs (144 ± 67 ACC/player/game) captured with the GPS-IMU devices, while the total number of ACCs with the video system was slightly lower (90,861 ACCs or 137 ± 62 ACC/player/game, *p* < 0.001). The number of DECs captured with the GPS-IMU devices (100,952 DEC or 131 ± 62 DEC/player/game) was also slightly inferior to that captured with the video tracking system (86,572 DECs or 131 ± 61 DEC/player/game, *p* < 0.001). The underestimation of data produced by the video system over the GPS-IMU was present in all variables, with differences between 5.0 and 21.9% (*p* < 0.001, Table 1) except for the number of ACCs at >4 m/s^2^, which was overestimated by −20.5 ± 11.1 (*p* < 0.001).

**Figure 1:**
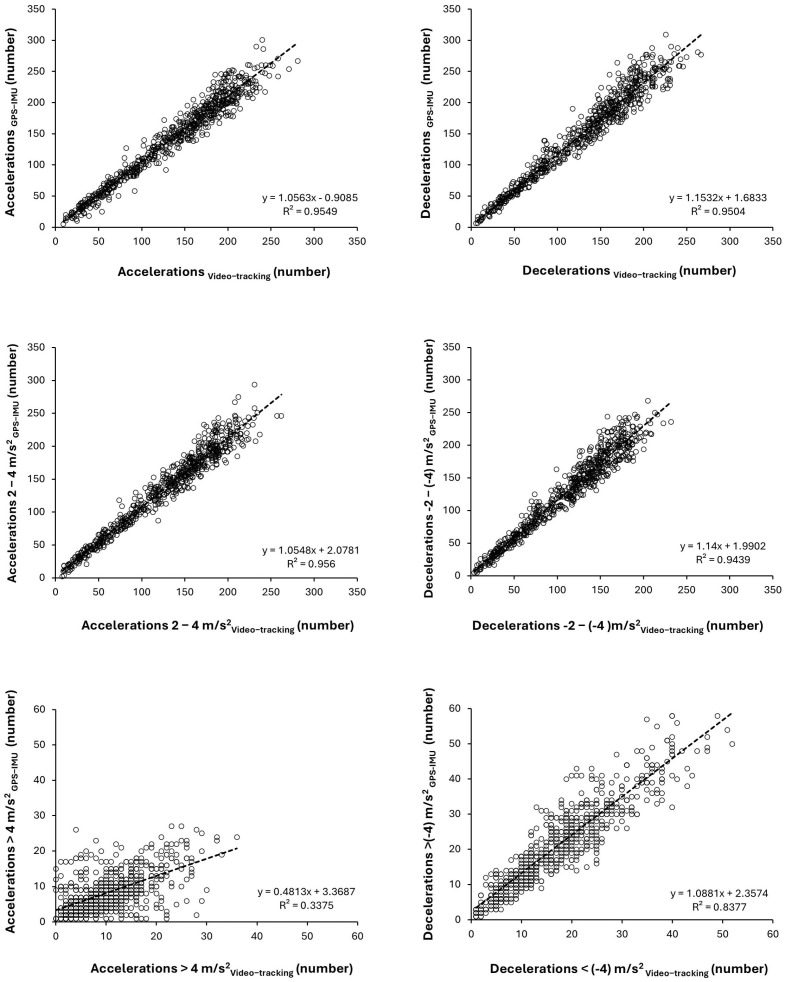
Correlations and regression equations for ACCs and DECs obtained with GPS-IMU devices (WIMU Pro; reference criterion) or calculated with the TRACAB Gen5 video tracking system coupled with Mediacoach software in 46 professional football players for 662 comparative match data. Each point represents the number of ACCs/DECs per player and per game.

The correlation analysis revealed very high agreement between the two systems for total ACCs (R^2^ = 0.954) and total DECs (R^2^ = 0.950), as well as for ACCs at 2–4 m/s^2^ (R^2^ = 0.956), DECs at −2–[−4] m/s^2^ (R^2^ = 0.943), and DECs < −4 m/s^2^ (R^2^ = 0.838). However, lower agreement was observed for ACCs at >4 m/s^2^ (R^2^ = 0.338). The same pattern of correlation was present for the ICC, which was very high for all variables (ICC > 0.90), with a moderate agreement for ACCs at >4 m/s^2^ (*p* < 0.001). Figure 1 depicts the graph of the correlation between systems for all variables under investigation, including regression equations.

**Figure 2:**
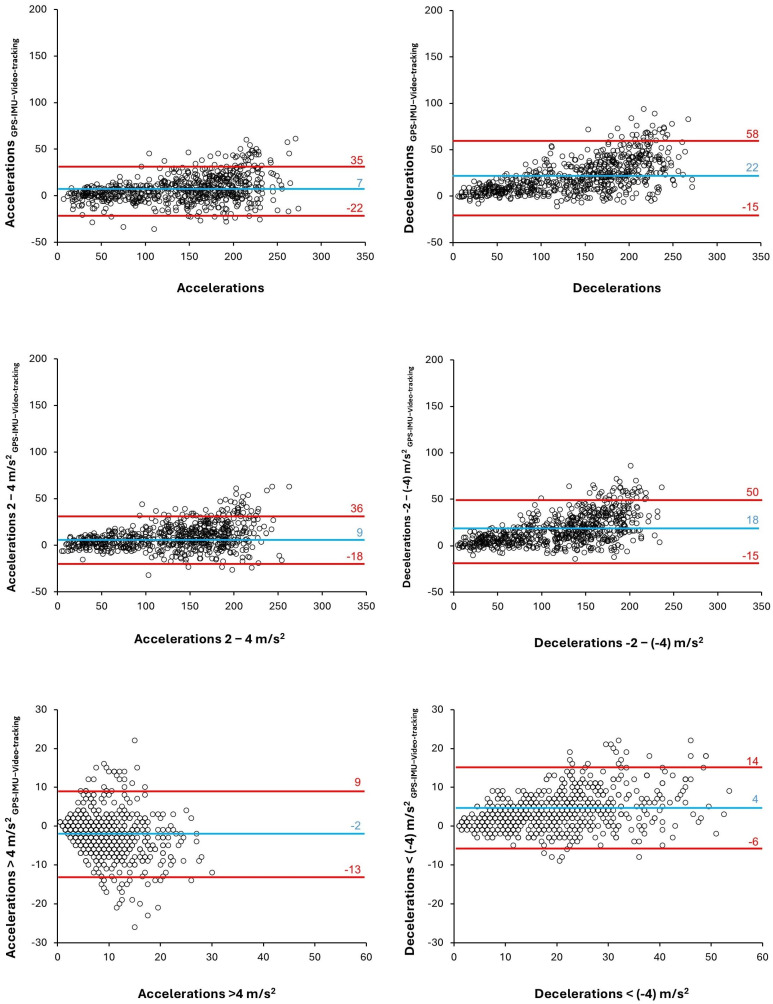
Bland–Altman plots for ACCs and DECs obtained with a GPS-IMU device (WIMU Pro) or calculated with the TRACAB Gen5 video tracking system coupled with Mediacoach software in 46 professional football players for 662 comparative match data. Each point represents the difference in ACCs/DECs per player and per game between the methods, taking the GPS-IMU device as the reference criterion.

The RMSE and SSE values indicated varying levels of error between the systems, with errors between 6.1 and 16.1 ACC/player/game and between 6.3 and 24.3 DEC/player/game (Table 2). The TEE was between 4.3 and 11.5 ACC/player/game and between 1.5 and 20.2 DEC/player/game. The SMB was between 0.1 and 0.6 for ACC and DEC variables. Last, the limits of agreement were between −2.2 and 9.0 ACC/player/game and between 3.9 and 21.7 DEC/player/game, with Bland–Altman plots further highlighting systematic underestimations by the video tracking system over the GPS-IMU device, particularly in total counts of ACCs and DECs (Figure 2).

## 4. Discussion

The aim of this study was to evaluate the validity of coupling the TRACAB’s Gen5 optical video tracking system with Mediacoach software for calculating ACCs and DECs during professional football matches. To achieve this, data on ACCs and DECs of varying intensities collected during official matches of *LaLiga* using this coupled method were compared to GPS-IMU devices, as these units are habitually considered as the reference value for the assessment of accelerometry data in team sports. The main findings of the current study revealed a very high agreement and error rates of small magnitude between the systems for total ACCs and DECs, as well as for subcategories of ACCs and DECs in the 2–4 m/s^2^ range. In contrast, a lower agreement was observed for ACCs exceeding 4 m/s^2^. Interestingly, the TRACAB’s Gen5-Mediacoach coupling consistently underestimated the total counts for both ACCs and DECs compared to the GPS-IMU data (it only overestimated ACC 4 m/s^2^), likely due to the difference in the rates of sampling. These results highlight the potential of optical tracking systems as a viable alternative to wearable devices for assessing ACC and DEC metrics in male professional footballers during competition. The high correlation values indicate that the TRACAB Gen5 Mediacoach system reliably tracks ACCs and DECs during gameplay and can identify players with higher or lower numbers of these actions across all intensity ranges. Despite the high levels of agreement, relatively small errors, and the availability of regression equations, the authors of this study do not recommend using video tracking data interchangeably with GPS-IMU data. In professional football, where accuracy is critical, even minor discrepancies in measuring players’ physical loads can lead to errors in managing both acute and chronic workloads.

This approach validates the integration of TRACAB Gen5 and Mediacoach as a reliable, non-invasive solution for measuring football players’ physical performance during competition, providing a practical alternative to wearable devices. The high agreement between the video system’s data and those from the GPS-IMU devices underscores the utility of this system for *LaLiga* teams, as it is the only competition that, to date, regularly provides data on physical load using TRACAB Gen5 and Mediacoach coupling. Thus, the present study recommends that Spanish professional football teams utilize Mediacoach as a convenient tool for evaluating physical performance metrics during official matches. However, caution is required when interpreting these data, particularly for high-intensity actions, and adjustments may be necessary to align them with GPS-IMU measurements for those teams comparing data from competition (registered by TRACAB Gen5 and Mediacoach) and training (habitually obtained with GPS-IMU devices).

A comparison with previous studies highlights the relative strengths and limitations of optical tracking systems in sports science. Our findings align with those by Pons et al. [18], as both studies report an acceptable level of agreement between tracking systems when measuring ACCs and DECs in professional football. Additionally, both studies found an underestimation of accelerometry data with the optical tracking systems with respect to GPS-IMU devices for the total number of ACCs/DECs and those from low-to-moderate intensity, suggesting that coupling TRACAB Gen5 and Mediacoach may offer a good alternative to wearable devices but still does not capture all ACCs and DECs performed during a match. Additionally, our study found lower levels of agreement for high-intensity accelerations (>4 m/s^2^), while TRACAB Gen5 and Mediacoach recorded a slightly higher number of ACCs at high intensities than the GPS-IMU devices, also coinciding with the study by Pons et al. [18]. Although it is important to note that Pons et al. [18] classified high-intensity actions as >3 m/s^2^ (which may influence the direct comparability of the results), the high coincidences between these two studies suggest that these two systems (GPS-IMU and video tracking) can be used to analyze players’ acceleration demands in professional football but with consideration that the video tracking underestimates overall acceleration data while it overestimates accelerations at high intensity.

Previous studies on the interchangeability of systems regarding running metrics observed positive correlations among variables between TRACAB and 10 Hz GPS devices, although TRACAB demonstrated higher values for all variables compared to GPS, especially in high-intensity actions and for larger distances, [15]. For instance, Linke et al. [13] reported the high validity of video tracking for running distances and its moderate validity for high-intensity actions. The current study aligns with these findings, demonstrating robust accuracy in detecting lower-intensity ACCs and DECs but a less strong capacity to reliably capture high-intensity events. Again, these findings suggest that TRACAB and GPS-IMU devices capture data with a high agreement, and they are able to detect players with higher vs. lower physical loads, but that they should not be used interchangeably [15,27] when evaluating running or mechanical metrics in professional football players.

This discrepancy between technologies may be due to the differing data sampling frequencies of the two systems, as TRACAB’s Gen5 operates at 25 Hz, whereas GPS-IMU devices sample inertial data at 1000 Hz, offering finer temporal resolution for rapid changes in velocity. The higher sampling rate of GPS-IMU devices likely leads to a more accurate detection of rapid changes in velocity, particularly for high-intensity movements, which may allow for a better assessment of ACCs and DECs. Another source of difference between these technologies is the nature of the data (estimated vs. measured). While video tracking systems calculate acceleration based on positional changes, GPS-IMU devices measure acceleration directly using four in-built accelerometers, along with gyroscopes, a magnetometer, and a barometer. This direct measurement of acceleration makes GPS-IMU devices the current reference standard for assessing accelerometry in football and other team sports. Additionally, both GPS-IMU and the TRACAB Gen5-Mediacoach systems employed algorithmic calculations to calculate ACCs and DECs, and they show differences in the smoothing or filtering processes applied to the data, which may cause the TRACAB Gen5-Mediacoach coupling to systematically count more events. Still, the algorithms and smoothing information of these systems are not publicly available, and we are unable to perform a deeper analysis to understand the nature of the differences.

Nevertheless, despite the high number of official matches analyzed (n = 57) and the consequently the high number of match data available for comparison (n = 662), the current agreement study has several limitations that should be acknowledged. First, the study was limited to a single team in *LaLiga*, potentially restricting the generalizability of the findings to other teams of *LaLiga* and other leagues. This is particularly relevant in this context, as the placement of the TRACAB multicamera system is unique in each stadium depending on the size of the pitch and the height of the supporting structures. Although TRACAB’s video tracking is fit to validly measure running parameters in all stadiums, a similar comparison in other stadiums of teams competing in *LaLiga* would be needed. Additionally, external factors such as the time of the match, lighting conditions, or camera obstructions, which can affect the accuracy of video tracking systems, were not controlled or analyzed. Furthermore, while the study focused on the agreement between systems, the physiological implications of discrepancies, particularly for high-intensity ACCs, remain unexplored.

The findings of this study have direct implications for coaches and fitness staff seeking to monitor and manage player workloads with greater precision. The validation of the TRACAB Gen5-Mediacoach system as a reliable tool for assessing ACCs and DECs during official matches offers teams a non-invasive alternative to IMU and GPS-based tracking. This allows practitioners to obtain valuable physical performance data without requiring players to wear additional equipment, which can be particularly beneficial in competitive environments where minimizing interference is crucial. However, given the observed discrepancies in high-intensity acceleration and deceleration counts, coaches should exercise caution when interpreting data from different tracking technologies. Adjustments using regression equations may be necessary to align optical tracking data with GPS-IMU-derived measures [15,27] for both running and accelerations data to ensure consistency in load monitoring across training and match contexts.

In conclusion, the TRACAB’S Gen5 optical tracking system coupled with Mediacoach software offers a valid method for assessing ACCs and DECs in professional football. However, it consistently registered lower values compared to the IMUs integrated into GPS devices, highlighting the need to take caution when interpreting data. Future research should focus on refining the calibration of optical systems for high-intensity movements and exploring their utility across diverse football environments. Integrating optical (during matches) and wearable systems (during training) may provide a comprehensive solution for performance monitoring in professional football.

## Figures and Tables

**Table 1:** Mean, standard deviation (SD), and range for the number of ACCs and DECs obtained with a GPS-IMU device (WIMU Pro) or calculated with the TRACAB Gen5 video tracking system coupled with Mediacoach software in 46 professional football players for 662 comparative match data. Data are ACC/decelerations per player and per game.

	GPS-IMU			Video Tracking					
Variable	Mean	SD	Range	Mean	SD	Range	Absolute Diff (%)	*p*	d
**Total ACCs**	144	67	5–301	137	60	9–281	5.0 ± 14.9	<0.001	0.47 small
**ACCs at 2–4 m/s^2^**	136	63	2–294	127	58	8–262	7.1 ± 17.0	<0.001	0.66 moderate
**ACCs at >4 m/s^2^**	9	6	1–27	11	7	1–36	20.5 ± 11.1	<0.001	0.39 small
**Total DECs**	152	72	6–309	131	61	6–267	16.6 ± 9.5	<0.001	1.17 moderate
**DECs at −2–[−4] m/s^2^**	131	62	5–268	113	53	4–232	15.8 ± 10.9	<0.001	1.08 moderate
**DECs at <−4 m/s^2^**	22	12	1–58	18	10	1–52	21.9 ± 13.2	<0.001	0.80 moderate

Data on GPS-IMU vs. video-tracking was compared with paired-samples *t* tests.

**Table 2:** Root mean square error (RMSE), standard error of estimate (SEE), typical error or estimate (TEE), standardized mean bias (SMB), coefficient of determination (R^2^), intraclass correlation coefficient (ICC), and limits of agreement (LoA) for the number of ACCs and DECs obtained with a GPS-IMU device (WIMU Pro) or calculated with the TRACAB Gen5 video tracking system coupled with Mediacoach software in 46 professional football players for 662 comparative match data. Data are ACCs/DECs per player and per game.

Variable	RMSE	SEE	TEE	SMB	R^2^	ICC	LoA
**Total ACCs**	16.1	16.1	11.4	0.1	0.954 <0.001	0.974 <0.001	6.8 ± 28.5
**ACCs at 2–4 m/s^2^**	16.3	16.3	11.5	0.6	0.956 <0.001	0.975 <0.001	9.0 ± 26.7
**ACCs at >4 m/s^2^**	6.1	6.1	4.3	0.0	0.338 <0.001	0.571 <0.001	−2.2 ± 11.1
**Total DECs**	28.5	28.6	20.2	0.3	0.950 <0.001	0.961 <0.001	21.7 ± 36.7
**DECs at −2–[−4] m/s^2^**	24.2	24.3	17.1	0.6	0.944 <0.001	0.959 <0.001	17.8 ± 32.3
**DECs at <−4 m/s^2^**	6.3	6.3	1.5	0.1	0.838 <0.001	0.902 <0.001	3.9 ± 9.6

## Data Availability

Data are contained within the article.
